# Vessel wall perforation mechanism of the excimer laser-assisted non-occlusive anastomosis technique

**DOI:** 10.1007/s10103-016-1950-7

**Published:** 2016-05-25

**Authors:** Jochem Bremmer, Tristan P. C. van Doormaal, Bon H. Verweij, Albert van der Zwan, Cornelius A. F. Tulleken, Rudolf Verdaasdonk

**Affiliations:** Brain Center Rudolph Magnus, Department of Neurosurgery, University Medical Center Utrecht, G.03.124, Heidelberglaan 100, 3584 CX Utrecht, The Netherlands; Brain Technology Institute, Utrecht, The Netherlands; Department of Physics & Medical Technology, Free University Medical Center Amsterdam, Amsterdam, The Netherlands

**Keywords:** Surgical anastomosis, Excimer laser, ELANA

## Abstract

The excimer laser assisted non-occlusive anastomosis (ELANA) technique is used to make anastomoses on intracerebral arteries. This end-to-side anastomosis is created without temporary occlusion of the recipient artery using a 308-nm excimer laser with a ring-shaped multi-fiber catheter to punch an opening in the arterial wall. Over 500 patients have received an ELANA bypass. However, the vessel wall perforation mechanism of the laser catheter is not known exactly and not 100 % successful. In this study, we aimed to understand the mechanism of ELANA vessel perforation using specialized imaging techniques to ultimately improve its effectiveness. High-speed imaging, high-contrast imaging, and high-sensitivity thermal imaging were used to study the laser wall perforation mechanism and reveal the mechanical and thermal effects involved. In vitro, rabbit arteries were exposed with the special designed laser catheter in a setup representative for the clinical setting, in which blood was replaced with a transparent UV absorbing liquid for visualization. We observed that laser vessel wall perforation was caused by explosive vapor bubbles tearing through the vessel wall, mostly within the first 20 of the total 200 pulses. Thermal effects were minimal. Unsymmetrical tension in the vessel wall inducing migration of the flap during laser exposure was observed in case of unsuccessful wall perforations. The laser wall perforation mechanism in the ELANA technique is primarily mechanical. Symmetric tension in the recipient vessel wall is essential and should be trained by neurosurgeons.

## Introduction

**Fig. 1:**
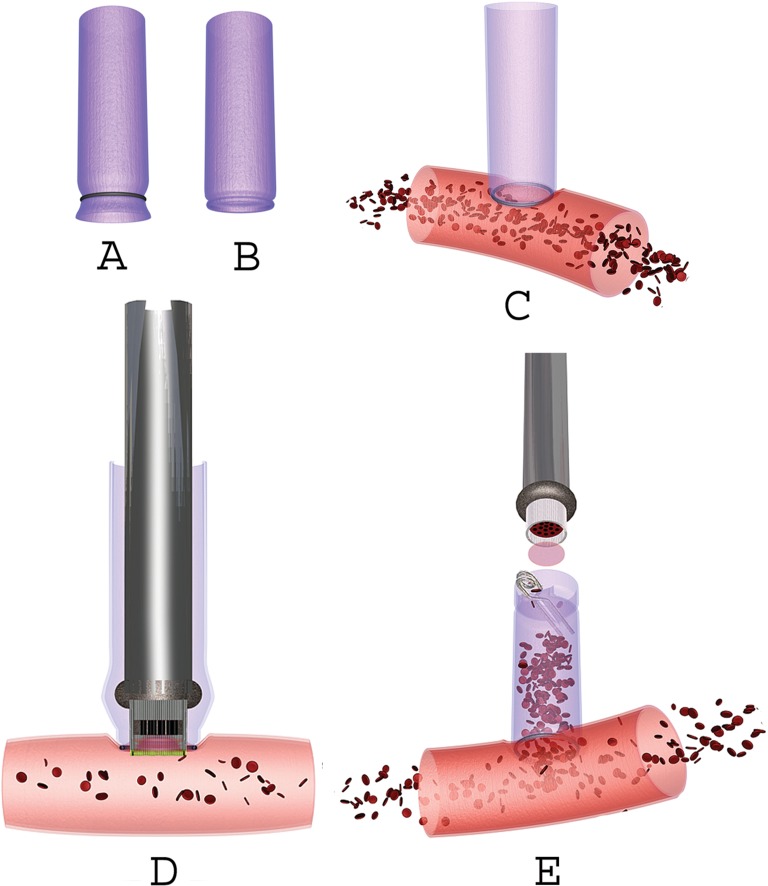
The excimer laser-assisted non-occlusive anastomosis (ELANA) procedure. **a**, **b** A 2.6- or 2.8-mm platinum ring is attached to the donor graft. **c** The bypass graft (with ring) is attached to the recipient artery. **d** Introduction of the catheter via the donor graft and activation of the laser. **e** The removal of the catheter with the arteriotomy flap. A temporary clip is applied on the donor vessel to avoid retrograde flow. NB: there is never interruption of the arterial flow in the recipient artery. Used with permission from [16]

**Fig. 2:**
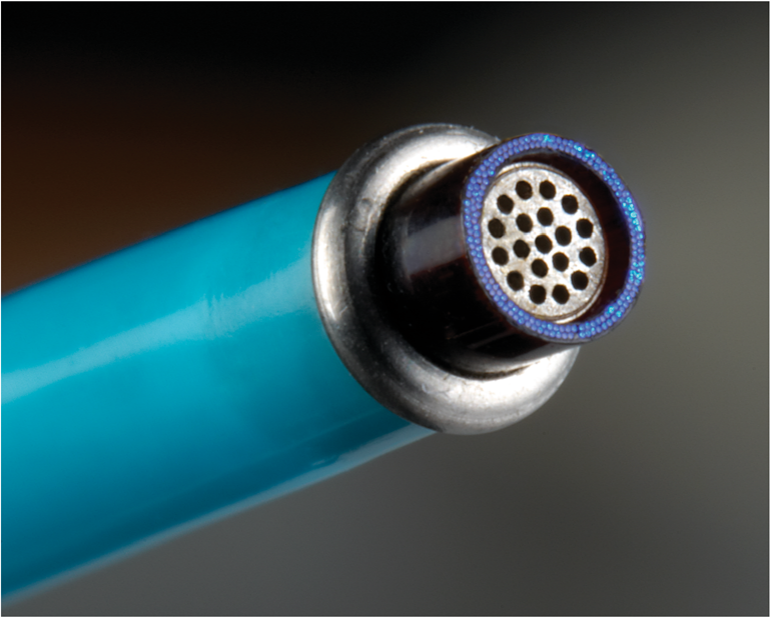
The ELANA catheter tip. The ELANA catheter 2.0® (ELANA B.V.®, Utrecht, The Netherlands) tip consists of 180 fibers (60-μm diameter) transmitting the laser light. These fibers are configured in two circles around a metal grid. The outer diameter of the ring of fibers is 2.0 mm. Vacuum suction is applied through the central lumen of the catheter to fixate the vessel wall on the metal grid in the tip

In very difficult-to-treat intracranial diseases, like giant aneurysms, tumors encasing cerebral arteries, or cerebral hemodynamic insufficiency caused by occluded cerebral arteries, it is sometimes indicated to create a high-flow bypass in the brain [1–4]. Temporary occlusion of proximal cerebral arteries while connecting an anastomosis harbors obvious significant ischemic risk. We therefore developed a technique that facilitates the creation of an anastomosis to a large brain artery without having to temporary occlude it: the excimer laser-assisted non-occlusive anastomosis (ELANA) technique [5, 6] (Fig. 1). The technique uses a specially designed laser catheter (Fig. 2) to punch a disc (the so-called “flap”) in the recipient artery. The ELANA technique has been used for the treatment of more than 500 patients and is CE and FDA approved.

The exact working mechanism of the ELANA technique is still unknown. It was only studied in parts, and the knowledge was based on the original research using the 308-nm excimer for laser angioplasty [7–10]. Although the ELANA technique showed excellent clinical results [1–4], it was shown that the full-thickness flap of recipient artery tissue was not retrieved in 12 % of all cases [11]. Not retrieving the flap seemed not to be associated with failure of the bypass [11] but is obviously a cause of severe discomfort during surgery for the surgeon because of theoretical embolization or flow hampering.

In this paper, we apply special imaging techniques to study the vessel wall perforation mechanism and catheter tip-tissue interaction to gain a better understanding of the mechanisms involved in the vessel wall cutting of the ELANA technique and to ultimately improve the flap retrieval rate.

## Materials and methods

### Laser settings and catheters

For all the imaging experiments, standard ELANA 2.0® catheters (ELANA B.V., Utrecht, The Netherlands, Fig. 2) were used with ∼100-ns pulses, energy set at 10 mJ, pulse repetition frequency at 40 Hz, and exposure time of 5 s (total 200 pulses). As laser source, a XeCl 308-nm CVX-300 laser was used (Spectranetics, Colorado Springs, CO, USA.). The tip of the catheter consists of two rings of 180 × 60-μm fibers resulting in a fluence of 2 J/cm^2^. The exact same settings are used when the system is used clinically. The ELANA catheters were visually checked for abnormalities after each experiment and replaced by a new catheter after 20 successive experiments.

### Animal materials

In this study, we used rabbit aortas as recipient artery [12]. The aortas were harvested at a consumption slaughterhouse (New Zealand white rabbits 2–4 kg). After harvesting the aortas, the vessels were put in separate containers filled with saline solution and then frozen at −10 °C. For each experiment in the model, a vessel was thawed in a saline solution at room temperature. The diameter of these vessels was approximately 4 mm, comparable to the diameter of the internal carotid artery in humans.

### Imaging

To study all the aspects of the ELANA wall perforation process, an imaging setup was used, which was adapted to the different experiments. Using a close-up lens, a mirror, and a video camera, a magnified view (around 4 mm across) of the laser wall perforation process could be achieved.

### Experiment 1

The basic interaction of the ELANA laser catheter with blood was studied using a high-speed imaging setup method that was previously described in other applications of excimer lasers in medicine [9, 10]. A 5-μs intense light flash was synchronized with the laser pulse from the excimer laser. In combination with a delay box, images were captured in sequence from 0 to 200 s with steps of 10 μs. Blood was replaced by water with a colorless UV absorber (oxybuprocaine-hydrochloride 6 gr/l resembling the absorption of 308-nm light in blood (OBP solution)). This way, the dynamics of the anticipated explosive vapor bubbles at the tip of the ELANA catheter could be visualized.

### Experiment 2

The next step was to visualize the vapor bubble formation while cutting through the recipient vessel wall. Therefore, ten squares (10 × 10 mm) were cut from a rabbit aorta. These tissue samples were positioned on pins with moderate stretch to provide a flat surface. The luminal side of the aorta was submerged in oxybuprocaine (OBP) solution. The ELANA catheter was placed perpendicular to the tissue sample with the catheter tip in direct contact with the tissue sample. The mechanism was visualized using the earlier described high-speed imaging setup. The cutting process was imaged from underneath the vessel as being inside the vessel with the catheter aimed toward the camera. This way, the progression of the vessel wall cutting could be followed from pulse to pulse.

### Experiment 3

To observe the formation/presence of potential gas bubbles and debris during the ablation process, the direct environment underneath the ablation site was imaged using a method based on Schlieren techniques [13]. This creates a very high-contrast enhancement, enabling the visualization of minor disturbances in fluid-like small particles, bubbles, and fluid motion induced by the ablation process. This experiment was repeated 15 times.

### Experiment 4

In addition, in an imaging setup using color Schlieren techniques [13], potential thermal effects were visualized during the wall perforation. The fluid layer underneath the vessel wall was observed closely with this highly sensitive thermal imaging setup. Small changes in refractive index due to heating were color coded, thereby visualizing the dynamics of heating and cooling during the ablation process. This experiment was repeated ten times.

### Experiment 5

**Fig. 3:**
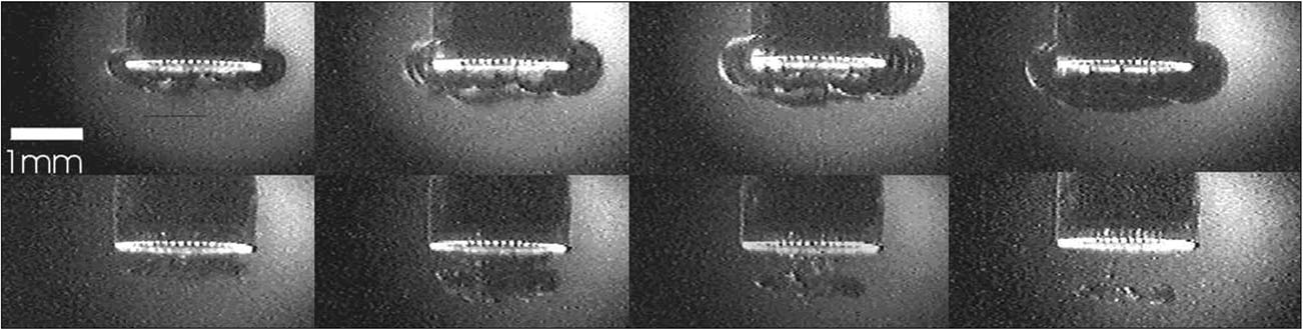
Typical example of formation of vapor bubbles underneath the fibers in the ELANA catheter. From the *top left corner* until the *bottom right corner*, the delay times of the photos taken after the laser pulse are 10, 30, 60, 90, 160, 220, 260, and 320 μs

Finally, to obtain a better understanding of the behavior of the vessel flap created during the laser exposure, an in vitro setup was made, representing the in vivo application of the ELANA technique. The wall perforation process was observed through a “window,” which was sutured in the artery directly opposite to the perforation site using a plastic sheet (Fig. 3). The prepared rabbit aorta was fixated in the model and stretched to 110 % compared to the length of the graft in a relaxed position. The aorta was filled with a transparent OBP solution (6 gr/l to mimic blood absorption at 308 nm) pressurized at 100 mm Hg using a gravity drip system. Subsequently, 80 ELANA anastomoses were created, using an experimental variant enabling a fast connection without sutures [14]. To standardize the force exerted with the ELANA catheter on the vessel wall during laser exposure, the catheter was attached to a laboratory scale (E200, Mettler-Toledo, Inc., Columbus, OH), using a custom-made construction. When the vacuum suction was activated, the catheter was continuously pushed down with 0.2 N. This is the optimum force for flap retrieval with one period of lasing [15]. The number of pulses needed for successful laser wall perforation was derived from the auto-fluorescence light from the tissue in front of each individual fiber induced by the 308-nm UV light. At the moment of tissue perforation, the fluorescence light disappeared.

## Results

### Experiment 1: vapor bubble in liquid

The high-speed imaging revealed a ring of rapidly expanding and imploding vapor bubbles at the tip of the ELANA laser catheter during laser exposure. The bubbles, formed at each individual fiber, merged to a torus or tube shape with a diameter of around 800 μm. The lifetime of the expanding and imploding bubble was 150 μs. A typical example of the bubble formation is shown in Fig. 3.

### Experiment 2: wall perforation

**Fig. 4:**
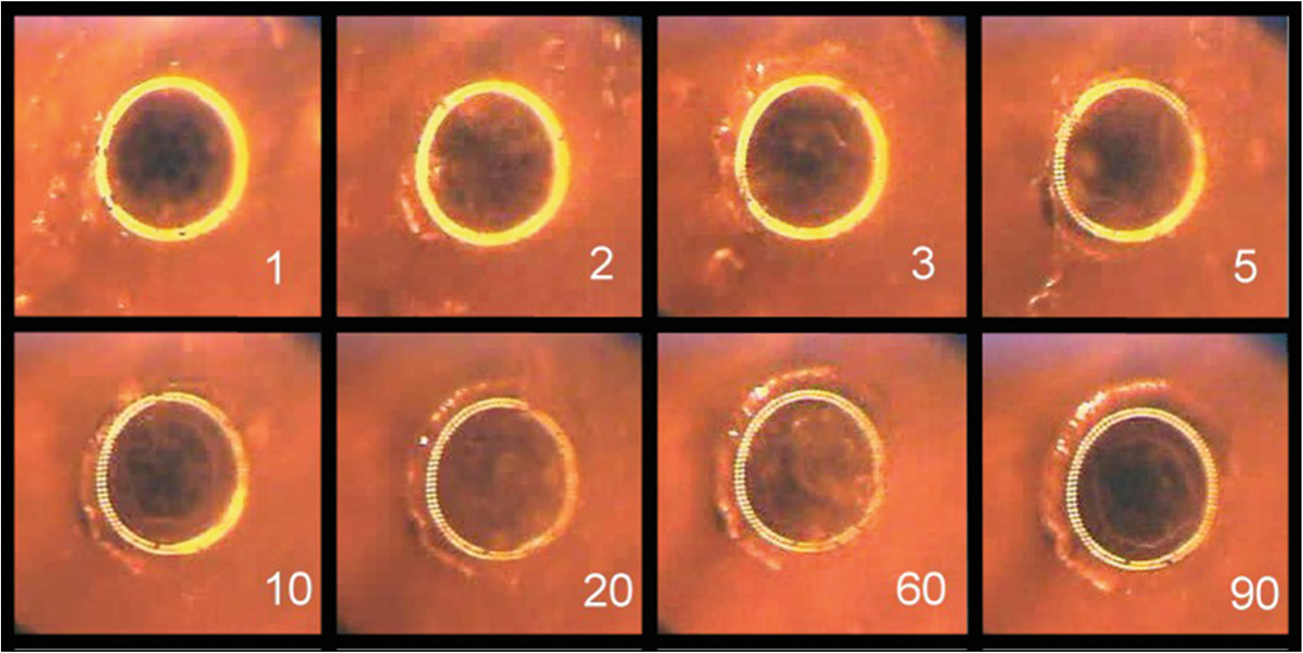
Example of vapor bubble formation during the perforation of the vessel wall with the ELANA catheter. The number of pulses is shown in the *lower right corner*. Before perforation, the 308-nm UV light induces auto-fluorescence light in front of the fibers. After about 60 pulses, most of the individual fibers can be distinguished

A typical example and description of vapor bubble formation, which was observed in every vessel wall perforation, are shown in Fig. 4. The vessel wall perforation was observed between the first 3 to 20 pulses which could be determined by the disappearance of the auto-fluorescence light from the tissue in front of each fiber. At ten pulses (0.25 s), on average, 75 % of the ring was totally perforated. During the ablation, a haze of minuscule debris was visible, obstructing the view on the suction holes in the center of the catheter lumen. This cloud of debris disappeared after approximately 80 pulses, and the suction holes became clearly visible through the semitransparent vessel wall. Between the residual 80 to 200 pulses, the appearance did not change significantly anymore.

### Experiment 3: ablation products

**Fig. 5:**
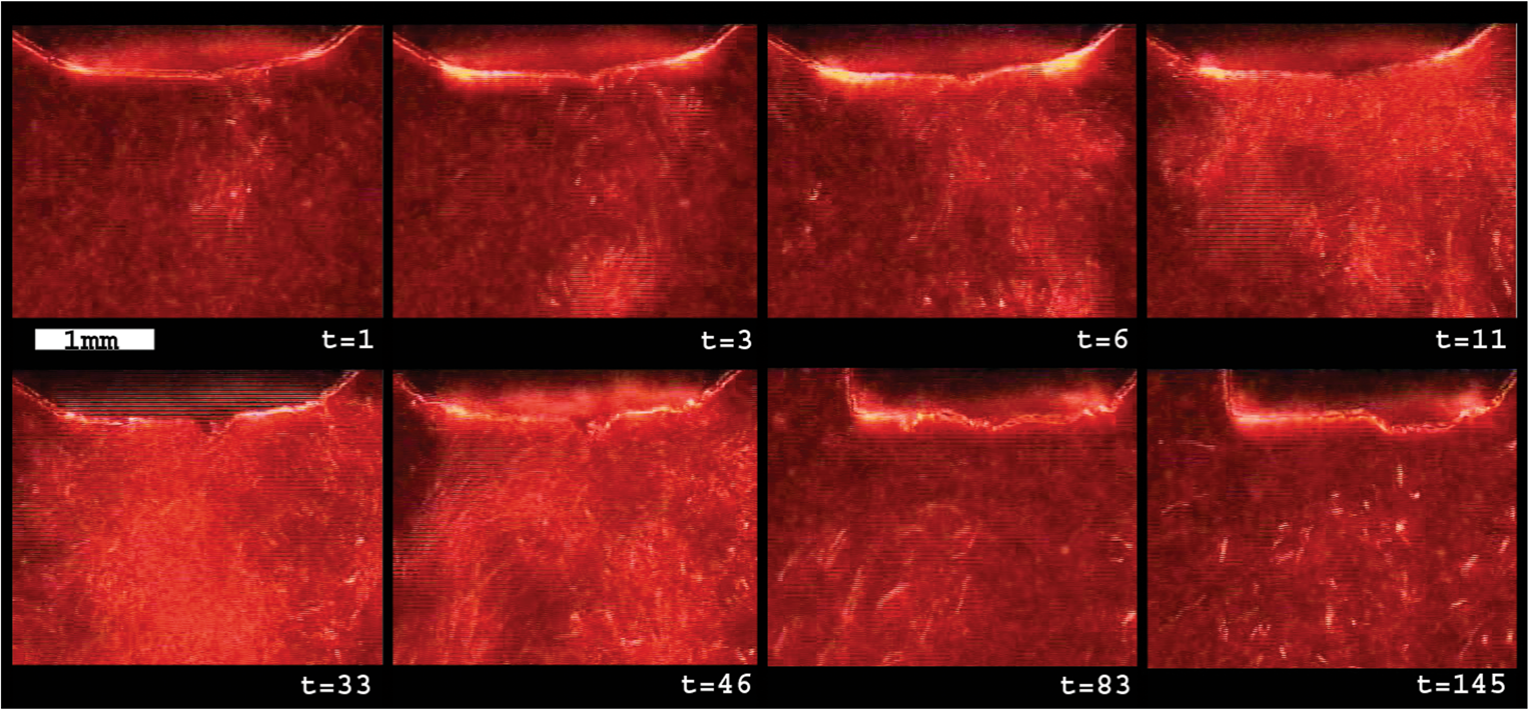
Example of high-contrast imaging based on Schlieren techniques. *t* number of pulses. Ablation debris can be seen as a cloud of particles estimated to be smaller than 20 μm. Gas bubbles were not observed. Around *t* = 83, the debris was vanished, indicating that no tissue was ablated anymore

The high-contrast images showed the turbulence in the fluid up to 4 mm underneath ablation site (Fig. 5). A dust cloud of very small particles (estimated to be smaller than 20 μm, based on the pixel resolution) is produced during the vessel wall perforation process revealing the mechanical component of the mechanism. The explosive vapor bubbles rupture the tissue structure and break the cell membranes releasing their content. No long-living ablation gas bubbles were observed, which are normally associated with high-temperature tissue dissociation. This is a strong indication that the temperatures do not reach far above the temperature of water vaporization (100 °C at 1 bar). A typical sequence of images during the ablation process is shown in Fig. 5. It shows that tissue perforation takes places within the first 20 pulses. After 80 pulses, the liquid underneath the activated catheter remains clear, indicating that the total ablation mechanism is finished.

### Experiment 4: thermal effects

**Fig. 6:**
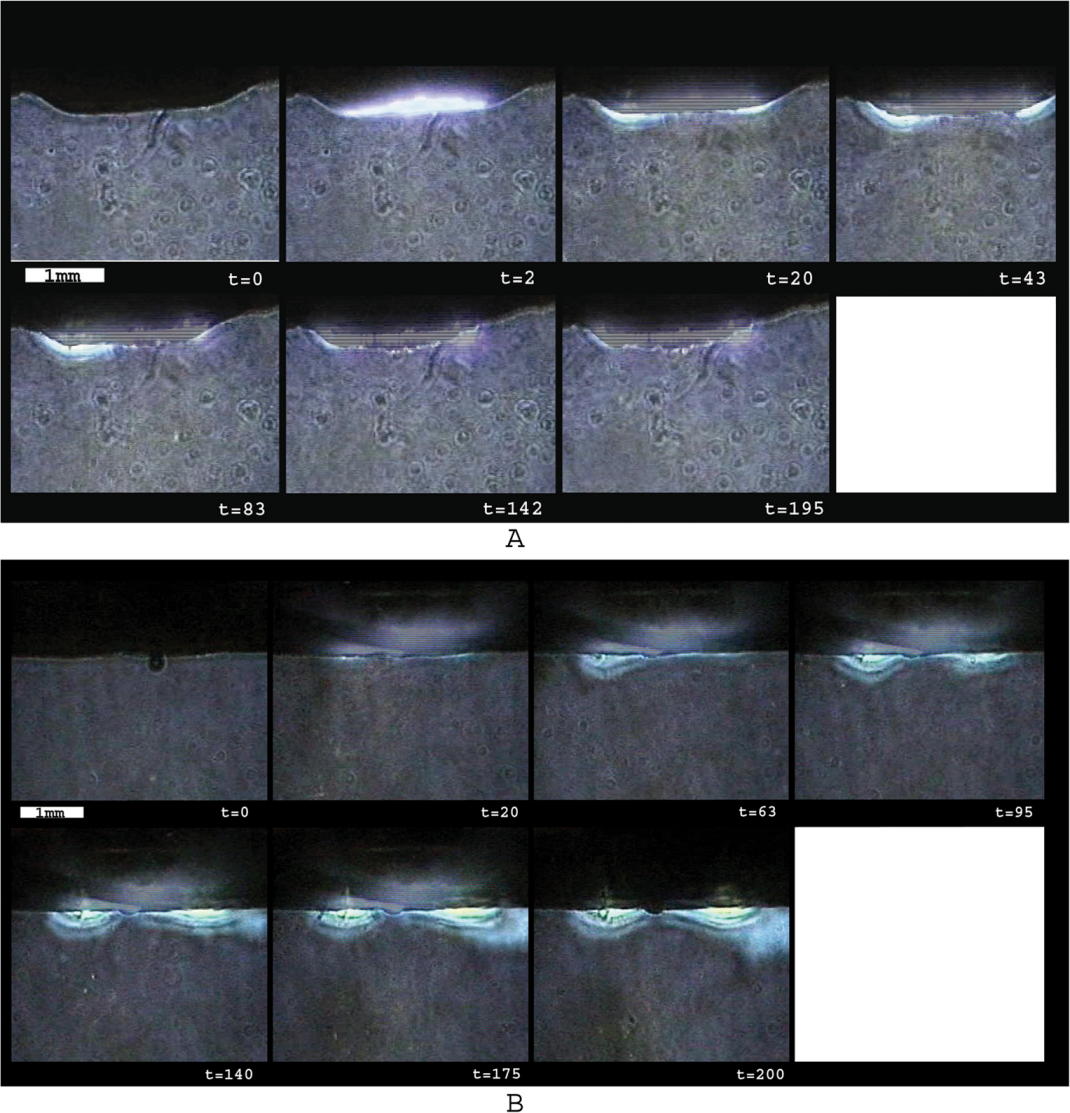
Thermal imaging (*t* = number of pulses). **a** Example of thermal imaging of a successful ELANA wall perforation. There is some thermal buildup from 20 to 40 pulses. After perforation (>80 pulses), the minimal thermal effects disappear. **b** Example of thermal imaging of a unsuccessful ELANA wall perforation (e.g., flap detachment from grid). Since there is no tissue perforation, the thermal energy accumulates inside the tissue and can only dissipate by conduction heating the water underneath the surface

The thermal effects were minimal. We observed the buildup of a small thermal zone underneath the tissue after the first 20–40 pulses, showing that the ablation process of the 308-nm laser starts basically as a thermal process. However, as soon as the tissue was perforated, no thermal effect was observed anymore. The severe turbulence induced by the explosive expanding and imploding vapor bubbles provided an effective dispersion of heat from the tissue surface. A typical example of heat formation during the laser wall perforation procedure is shown in Fig. 6a. However, when tissue perforation was unsuccessful (Fig. 6b), e.g., due to detachment of the vessel wall from the catheter grid, the thermal energy accumulates inside the tissue and can only dissipate by conduction, thus heating the water underneath the surface.

### Experiment 5: flap retrieval

**Fig. 7:**
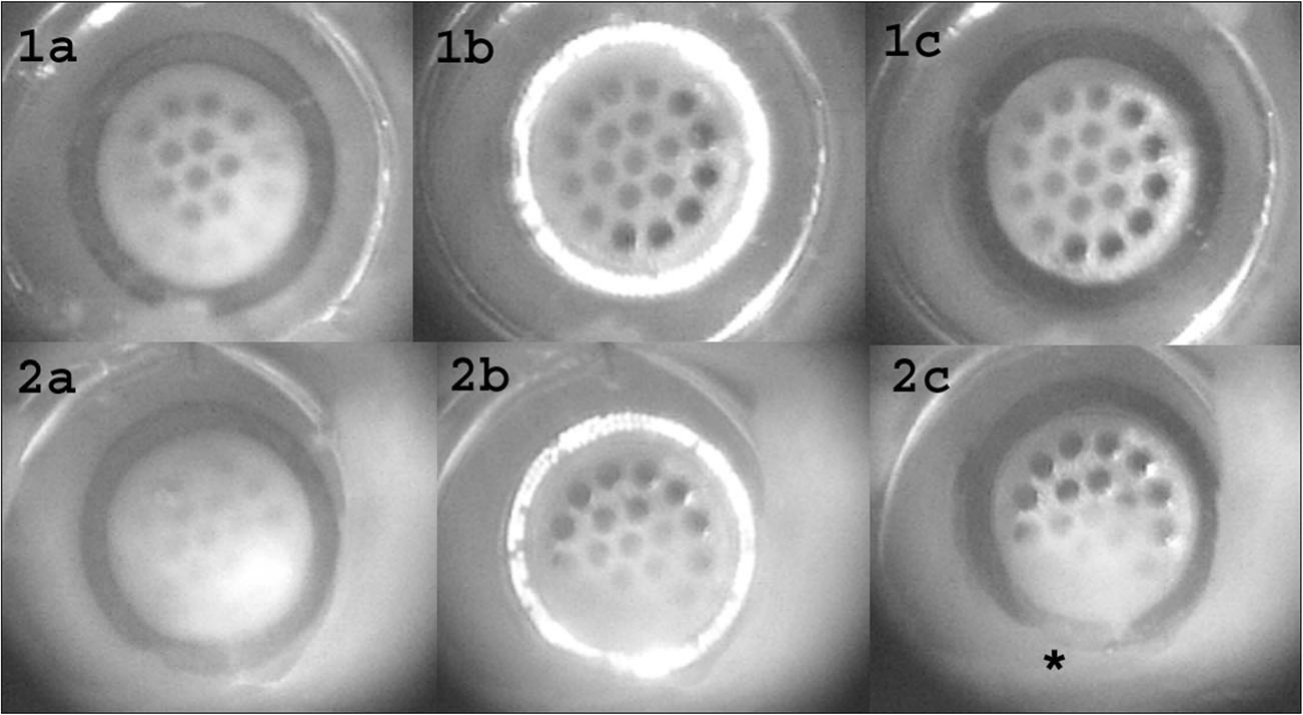
Video imaging of the laser wall perforation process. *1a–c* Example of successful laser wall perforation: *1a* last frame before laser activation, *1b* laser wall perforation, with the presence of fluorescence from the tissue in front of the fibers; and *1c* complete vessel wall perforation. *2a–c* Example of unsuccessful laser wall perforation: *2a* last frame before laser activation, *2b* laser wall perforation, and *2c* incomplete vessel wall perforation after 200 pulses. Notice that the wall perforation is not complete at the *asterisk* after 200 pulses

A typical example of the imaging during the vacuum suction phase and a successful laser procedure is shown in Fig. 7 (1a–c). When the vacuum was activated, the vessel wall was pulled against the grid in the lumen of the catheter. The degree of contact between the vessel wall and the grid varied; however, this was unrelated to successful flap retrieval. When the laser was activated, the vessel wall was totally perforated and the flap was retrieved in 70 of the 80 anastomoses (88 %). In these anastomoses, complete perforation was already achieved after mean 27 pulses (range 10–40 pulses).

However, in ten (12 %) of the laser procedures, a flap could not be retrieved within the normal number of laser pulses (Fig. 7 (2a–c)). We observed two different types of laser wall perforation failure. In two of the ten failed procedures, after a few laser pulses, the flap moved to one side, loosened, and then detached completely from the metal grid. Direct contact between the laser fibers and the vessel wall was lost, and therefore, the laser could not perforate the remaining tissue. In eight of the ten failed procedures, the flap was perforated on one side, and due to the internal stress in the flap, the remaining part was pulled to the opposite site. The flap did stay attached to the grid, but the laser was not able to perforate a complete circle out of the vessel wall in the given 200 pulses.

## Discussion

The exact laser wall perforation mechanism of the ELANA technique was unclear until now. In this study, we used advanced imaging techniques to obtain a better understanding of the vessel wall perforation mechanism with the ELANA catheter.

The high-speed imaging, high-contrast imaging, and thermal imaging of the vessel wall perforation showed rapidly expanding and imploding vapor bubbles. The water in the tissue, either intracellular or extracellular, is instantly turned to water vapor, creating the explosive bubbles that rupture the cell and tissue bindings. The content of the cells is dispersed as very small particles in the surrounding area. Within a few pulses, the vapor breaks through the thin vessel wall and the vapor can freely expand in the liquid. Remarkable was the absence of a significant thermal effect and the associated long-living gas bubbles which are typically created as ablation product with high-temperature (>200 °C) ablation [9]. This is most likely caused by the effective dispersion of heat in the turbulent liquid after the condensation of the vapor (bubble implosion) and a lack of temperature buildup in the thin arterial wall. We can therefore conclude that the perforation mechanism of the ELANA catheter is mainly based on water vaporization in combination with explosive vapor expansion (mechanical effect).

Although our observations are mostly qualitative, they are reproducible and provide sufficient insight to draw conclusions on the mechanism of action of the ELANA catheter. We can also confirm that there are no long-living gas bubbles formed and that, based on the image resolution, the size of the debris is not larger than 20 μm.

In the clinical simulation experiment, we observed that ten flaps (12 %) were not retrieved. This is in line with all previous in vitro and clinical studies that show, besides excellent technical and patient results, an incomplete vessel wall perforation with the ELANA catheter in 10–16 % of cases when lased with one episode of 5 s with ∼100 ns per pulse at 10 mJ and 40 Hz pushing the catheter down with 0.2 N [1, 2, 11, 15, 16]. We described earlier that, in most cases, a not-retrieved flap is not hampering bypass flow and the anastomosis can still be used [11]. However, numbers are relatively low, and in a few cases, the remaining flap did potentially obstruct bypass flow, resulting in abandoning of the anastomosis. We therefore believe that we should aim at 100 % flap retrieval.

The results of this study show the importance of symmetric tissue tension within the ELANA ring before starting the laser. In eight out of the ten missed flaps, the missed flap was still attached to the grid after lasing before retrieval of the catheter from the anastomosis. This flap could potentially be retrieved with a second period of laser activation. This theory was confirmed in an earlier study [15]. In this empirical study by van Doormaal et al., 2280 anastomoses were created in vitro, varying laser energy, episodes, and application pressure. It was concluded that the flap retrieval rate of the ELANA anastomosis technique can be optimized to 100 % by setting the laser energy at 15 mJ. However, it was later shown in an in vivo study that this setting damages the adjacent endothelium for mean 70 %, while 10 mJ leaves the endothelium for mean 90 % completely intact [17]. As alternative, a second laser episode of 10 mJ resulted in an increase in flap rate from 86.7 to 98.3 % [15]. It was also shown in this study that 0.1–0.2 N of pressure should be applied on the catheter during lasing, because a higher pressure resulted in a significantly lower flap rate.

Combined with the results of the current study, the importance to facilitate a dedicated microsurgical training period for every surgeon who wants to use the ELANA technique is evident. Perfectly placed microsutures creating a symmetric recipient vessel wall within the ring combined with adequate pressure manually exerted on the catheter are essential. As next step, new feedback mechanisms should be developed to evaluate the symmetry of the suturing and vessel wall tension, both for training as in the real surgical situation. However, the real in vivo situation often differs from ideal in vitro circumstances. Human cerebral arteries can be sclerotic, they can vary in vessel wall thickness, and sometimes, a perfect perpendicular catheter position is difficult to achieve because of anatomical reasons. Therefore, in the near future, flap-rate improvement with training and a second laser activation should be evaluated in a patient study.

## Conclusion

We conclude that the laser wall perforation mechanism in the ELANA technique is primarily mechanical, based on explosive tissue water vaporization with explosive bubble formation contributing to the tearing of tissue in direct contact with the catheter tip. A symmetric recipient vessel wall within the ELANA ring and direct laser fiber-tissue contact during the laser wall perforation are essential for successful flap retrieval and should be trained by neurosurgeons.
